# Editorial: Exploring health disparities in black communities: historical perspectives, present challenges, and future directions

**DOI:** 10.3389/fpubh.2026.1787163

**Published:** 2026-02-04

**Authors:** Bruno Bonnechère, Kayode Ijadunola, Elias Ali Yesuf, Judite Blanc, Yashendra Sethi

**Affiliations:** 1 Faculty of Rehabilitation Sciences, REVAL Rehabilitation Research Center, Hasselt University, Diepenbeek, Belgium; 2 Technology-Supported and Data-Driven Rehabilitation, Data Sciences Institute, Hasselt University, Diepenbeek, Belgium; 3 Department of PXL – Healthcare, PXL University of Applied Sciences and Arts, Hasselt, Belgium; 4 Elizade University, Ondo, Nigeria; 5 Jimma University, Jimma, Ethiopia; 6 Leonard M. Miller School of Medicine, University of Miami, Miami, FL, United States; 7 Subharti Medical College, Meerut, India

**Keywords:** black communities, chronic disease, health disparities, health equity, historical injustices, maternal health, mental health, public health

The enduring reality of health disparities affecting Black communities worldwide remains one of the most profound moral and scientific challenges of our time. These inequities do not arise from random variation or biological destiny, but from a complex tapestry of historical injustices, structural racism, socio-economic forces, policy choices, and persistent barriers to equitable health access and outcomes. In this Frontiers in Public Health Research Topic, “*Exploring Health Disparities in Black Communities: Historical Perspectives, Present Challenges, and Future Directions*,” a comprehensive, multidisciplinary platform is on offer to highlight these issues with nuance, rigor, and urgency.

Black History Month and broader movements for racial justice compel us to reflect not just on the existence of disparities, but also on the roots and trajectories of inequity. This topic serves as both a scholarly milestone and a call to action to deepen our understanding of how past and present forces shape health outcomes in Black communities and to envision future pathways that prioritize equity, dignity, and justice.

To understand contemporary health disparities, we must first reckon with history. Black communities have endured centuries of exploitation, discrimination, and exclusion in healthcare and biomedical research. In the United States, abhorrent episodes such as the Tuskegee Syphilis Study and the exploitation of enslaved women for surgical experimentation exemplify how racism became embedded in medical science and public health practice. These events contribute to a legacy of mistrust toward healthcare systems that cannot be ignored when examining current patterns of care, engagement, and health behavior.

The topic foregrounds this critical historical dimension, emphasizing how historical injustices and segregation have shaped not only distrust, but also deeply unequal access to resources, opportunities, and life-saving care (Myles). By centering this context, contributors elucidate a central truth: health disparities are not merely biological or behavioral phenomena, but social and political: they are imprints of inequity (Walelo and White Whilby; Mehrtash).

At the heart of racial health disparities lie the social determinants of health: the conditions in which people live, work, learn, and age. Income inequality, housing and environmental quality, educational opportunities, food access, employment conditions, neighborhood safety, and systemic discrimination exert powerful influences on health outcomes (Woodhouse et al.; Contreras et al.). These determinants do not impact all communities equally; they are structured by historical patterns of segregation, resource allocation, and policy bias that disproportionately harm Black communities.

Contributions within this issue have explored how socioeconomic disadvantage intersects with racial categorization to shape exposure to risk and access to protective resources. Authors highlight that disparities in chronic conditions such as hypertension, diabetes, and obesity—often prevalent in Black populations—reflect not only individual health behaviors but long-standing inequities in opportunities for health and wellbeing (Littleton et al.; Prochnow et al.).

Importantly, this Research Topic also advances a relational understanding of health: one that considers how policies and systems—from zoning laws to labor markets to school funding—weave the fabric of health disadvantage long before a condition is diagnosed in a clinic (Walelo and White Whilby; Contreras et al.).

The burden of chronic disease in Black communities remains disproportionate. Contributors analyze how a confluence of genetic, environmental, institutional, and systemic factors contributes to higher prevalence and poorer outcomes for conditions including cardiovascular diseases, diabetes, and metabolic disorders (Woodhouse et al.). But beyond the clinical metrics lie experiences of care influenced by bias, access barriers, and differential treatment quality (Myles).

Equally critical is the attention this issue pays to mental health, a domain too long marginalized in discussions of racial health disparities. Contributors examine barriers to mental health care, cultural stigma, and the systemic neglect of mental wellbeing in policy planning and resourcing (Meyer et al.). These explorations reinforce the insight that health equity must encompass emotional and psychosocial dimensions of wellness.

One of the starkest indicators of persistent inequity is the disproportionate rates of maternal and infant mortality affecting Black women globally. Research in this issue analyses the interplay of clinical care, access to prenatal services, socio-economic stressors, and culturally competent practices that may mitigate such preventable deaths (Amekpor et al.). By foregrounding maternal and infant health, the topic underscores that disparities begin at life's earliest stages and reflect gendered and racialized vulnerabilities that demand urgent policy and community responses (Contreras et al.).

Understanding disparities is only part of the mission; translating that knowledge into equitable policy and practice is paramount. The Research Topic includes analyses of health policy, advocacy efforts, and systemic reform. Contributors evaluate legislative strategies, health system reforms, and community-driven public health innovations that seek to disrupt patterns of inequity (Bonnechère).

Community-based interventions and culturally tailored health campaigns serve as promising exemplars (Tanywe et al.). These innovations, often rooted in local knowledge and community leadership, offer pathways to bridge gaps in access, trust, and outcomes. Such work reminds us that equity is cultivated not only in academic analysis but through sustained engagement with affected communities.

Finally, the topic emphasizes the indispensable role of cultural competence in healthcare (Amekpor et al.; Bonnechère). Addressing implicit bias, improving provider–patient communication, and integrating anti-racist curricula in health professions training are critical steps toward equitable care. These efforts acknowledge that healthcare systems must transform internally as well as externally to serve diverse populations effectively.

The breadth and depth of work presented in this Research Topic reflect a shared commitment among scholars, practitioners, and community leaders to move beyond description toward tangible change. The editorial draws attention to future directions that include the following:

- Interdisciplinary research that links historical analysis, social science, and epidemiological data to unpack complex determinants of health inequities
- Policy experimentation that tests and scales equitable interventions in healthcare access, quality, and outcomes
- Community partnership models that elevate the voices and expertise of Black communities in designing, implementing, and evaluating health initiatives
- Global perspectives that recognize the interconnectedness of racial health disparities across diasporic and international contexts

This agenda urges a holistic approach that dismantles structural barriers while building new systems centered on fairness, dignity, and shared prosperity.

In a world where racial inequities persist despite decades of advocacy, research, and policy endeavors, this Research Topic stands as a testament to the power of collaborative inquiry and sustained commitment. It invites its readers—researchers, practitioners, policymakers, and community members alike—to engage deeply with the realities of health disparity and to act with urgency, courage, and empathy.

The journey toward equity begins with understanding, but it cannot end there. The work ahead demands that we listen, learn, and co-create solutions that honor the health and dignity of Black communities everywhere.

